# Publication trends on the posterior cruciate ligament over the past 10 years in PubMed: Review article

**DOI:** 10.1016/j.amsu.2020.05.040

**Published:** 2020-05-29

**Authors:** Sholahuddin Rhatomy, Dwikora Novembri Utomo, Heri Suroto, Ferdiansyah Mahyudin

**Affiliations:** a Doctoral Program, Faculty of Medicine, Universitas Airlangga, Indonesia; b Department of Orthopaedic and Traumatology, Faculty of Medicine, Universitas Airlangga, Indonesia

**Keywords:** Posterior cruciate ligament, PCL, Publication, PubMed, Database search, Knee

## Abstract

**Background:**

The posterior cruciate ligament (PCL) is one of the rare operated ligament of the knee. Details on the top journals, universities, and authors on the topic would be helpful to identify the sources of information for clinical and research queries as well as to observe trends for future research and identify universities/authors of particular interest for training or to follow their research.

**Purpose:**

To consolidate information from PubMed on the PCL from 2009 to 2019, spanning 10 years.

**Study design:**

Cross-sectional study.

**Methods:**

A search of the PubMed database was conducted for the PCL, and 593 articles published over the past 10 years were analysed for further details. These details included the number of publications per year, top 10 journals publishing on the PCL, top 10 first authors publishing articles on the PCL.

**Results:**

The top journal and top author in all position publishing on the PCL were Knee surgery, sports traumatology, arthroscopy and Robert F. LaPrade, respectively. The most articles published by a first author were by Yong Seuk Lee. The United States was the most published country, and 4 of the top 10 affiliations were from this country.

**Conclusion:**

Mining the data on the PCL in PubMed produced useful information about good sources of publications on this topic, including authors/journals that could be followed. The strength of their association with other authors could potentially indicate co-workers, common research interests, and collaborative studies.

## Highlights

• Publication Trends.
• Posterior Cruciate Ligament.
• Over the Past 10 Years.

## 1 Introduction

PubMed was first released in January 1996 as an experimental database under the Entrez retrieval system with full access to MEDLINE®. The word “experimental” was dropped from the Web site in April 1997, and on June 26, 1997, a Capitol Hill Press conference officially announced free MEDLINE access via PubMed. PubMed searches were approximately two million for the month of June 1997 while current usage typically exceeds three million searches per day [1].

Posterior cruciate ligament (PCL) tears are about 3% of outpatient knee injury and 38% of acute traumatic knee hemarthrosis. This injury rarely occur in isolation, and up to 95% of PCL tears occur in combination with other ligamentous tear. With increasing on sports activities, this injury will increase in the future. PCL rupture are increasingly of morbidity and decreased function, persistent instability, pain, impaired function and the development of degenerative joint disease [2,3].

A better understanding of the anatomy and biomechanics of PCL has emerged. It leads to improved surgical techniques and rehabilitation program. However, there is still controversy about treatment decision whether operative or non-operative, rehabilitation program and optimal outcome. Studies with longterm follow-up are still low in number. This study is aimed to consolidate knowledge about PCL journal publications in the PubMed from 2009 to 2019.

## 2 Methods

The terms used to search in PubMed were “posterior cruciate liga-ment” [MeSH] OR “PCL”. The search resulted in a total of 13,704 articles from 1915 to 2020. Further, 9132 articles between 2009 until 2019 were included. After screening of the titles, abstracts and full-text assessment, 593 articles were eligible for further analysis in this study. We have registered our study at researchregistry with unique identifying number (UIN) reviewregistry894 [4].

All types of studies were included: human and animal studies including molecular/laboratory study, animal study, cadaveric study, imaging study, clinical study, systematic review and meta-analysis. All types of articles were sought in all languages. Excel (Office 365 for Mac; Microsoft) was used to create a database, analize the data and visualize the data.

## 3 Results

**Fig. 1:**
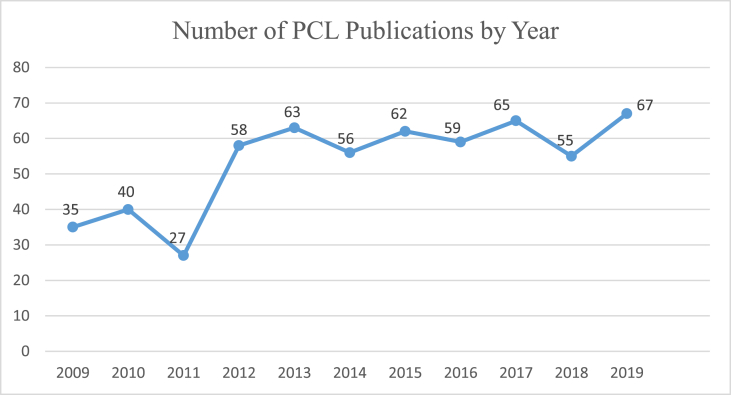
Number of PCL publications by year.

Fig. 1 shows the yearly distribution of publications. The average number of published articles per year was 59. The significant increment in publications occur in 2011–2012. In 2009, the number publication was 35 and it was increased for almost twice in 2019.

### 3.1 Journals

Table 1 shows the top 10 journals publishing on the PCL. A total of 593 journals have published on the given search terms from 2009 to 2019. Knee surgery, sports traumatology, arthroscopy was the leading publisher on this topic, with 77 articles published from 2009 to 2019. The top 10 journals published 46,6%, while the top 5 journals (Knee surgery, sports traumatology, arthroscopy; Arthroscopy: the journal of arthroscopic & related surgery; The American Journal of Sports Medicine; The journal of knee surgery; and Archives of orthopaedic and trauma surgery) published 33% of all articles on the PCL in the study period.

**Table 1:** Top 10 journals publishing on the PCL.

No	Journal name	Number of Publications	Percentage (%)
1	Knee surgery, sports traumatology, arthroscopy	77	12,9
2	Arthroscopy: the journal of arthroscopic & related surgery	40	6,7
3	The American journal of sports medicine	35	5,9
4	The journal of knee surgery	24	4
5	Archives of orthopaedic and trauma surgery	23	3,8
6	Arthroscopy techniques	23	3,8
7	The Knee	19	3,2
8	Orthopedics	15	2,5
9	Current reviews in musculoskeletal medicine	12	2
10	Orthopaedic journal of sports medicine	11	1,8

### 3.2 Authors

Table 2 shows the top 10 first author publishing on the PCL. A total 4 authors were affiliated from South Korea.

**Table 2:** Top 10 first author on PCL research and publications.

No	Orthopaedic Center	Investigator	Number of Publications
1	Department of Orthopaedic Surgery, Bundang Hospital, Seoul National University College of Medicine, 166 Gumi-ro, Bundang-gu, Seongnam, Gyeonggi, 463-707, South Korea	Yong Seuk Lee	9
2	Department of Sports Medicine and Orthopaedics, Geisinger Health System, Danville, Pennsylvania, USA.	Gregory C Fanelli	8
3	Department for Trauma Surgery and Sports Traumatology, Academic Hospital Feldkirch, Feldkirch, Austria	Michael Osti	6
4	Department of Orthopaedic Surgery, Arthroscopy and Joint Research Institute, Yonsei University College of Medicine, Seoul, South Korea	Sung-Jae Kim	6
5	Faculdade de Cie^ncias Médicas da Santa Casa de Misericórdia de São Paulo (FCMSCSP), Departamento de Ortopedia e Traumatologia, São Paulo, SP, Brazil	Ricardo de Paula Leite Cury	5
6	Department of Radiology, Haraldsplass Deaconess Hospital, Bergen, Norway	Anagha P Parkar	4
7	Center for Musculoskeletal Surgery, Charite - Medical University of Berlin, Germany	Clemens Gwinner	4
8	Department of Radiology, Kangbuk Samsung Hospital, Sungkyunkwan University School of Medicine, Seoul, South Korea	Hee Jin Park	4
9	Department of Orthopaedic Surgery, Samsung Medical Center, Sungkyunkwan University School of Medicine, Seoul, South Korea	Jin Hwan Ahn	4
10	Steadman Philippon Research Institute, Vail, Colorado, USA	Jorge Chahla	4

Table 3 shows the top 10 authors with the most publications in all position. Robert F. LaPrade has published the most number with 30 articles, which is constituted 5% of all articles published on this topic.

**Table 3:** Top 10 authors with the most publications in all position.

No	Orthopaedic Center	Investigator	Number of Publications
1	Steadman Philippon Research Institute, Vail, Colorado, USA	Robert F. LaPrade	30
2	Department of Orthopaedic Surgery, Bundang Hospital, Seoul National University College of Medicine, 166 Gumi-ro, Bundang-gu, Seongnam, Gyeonggi, 463-707, South Korea	Yong Seuk Lee	16
3	Steadman Philippon Research Institute, Vail, Colorado, USA	Lars Engebretsen	13
4	Department of Orthopaedic Surgery, Samsung Medical Center, Sungkyunkwan University School of Medicine, Seoul, Korea	Jin Hwan Ahn	10
5	Department of Sports Medicine and Orthopaedics, Geisinger Health System, Danville, Pennsylvania, USA	Gregory C Fanelli	8
6	Department of Orthopedic Surgery and Sports Medicine, Mayo Clinic, 200 First Street SW, Rochester, MN, 55905, USA	Bruce A. Levy	8
7	Department of Orthopaedic Surgery, Samsung Medical Center, Sungkyunkwan University, School of Medicine, #81, Irwon-Ro, Gangnam-gu, Seoul, 135-710, South Korea	Joon Ho Wang	8
8	Oslo Sports Trauma Research Center and Department of Orthopedic Surgery, Akershus University Hospital, University of Oslo, 1478, Lorenskog, Norway	Asbjorn Aroen	7
9	Department for Trauma Surgery and Sports Traumatology, Academic Hospital Feldkirch, Carinagasse 47, 6800, Feldkirch, Austria	Michael Osti	7
10	Center for Joint Diseases and Rheumatism, Department of Orthopaedic Surgery, Kyung Hee University Hospital at Gangdong, 892 Dongnam-ro, Gangdong-gu, Seoul, South Korea	Sang Hak Lee	7

### 3.3 Most cited/Important articles

Table 4 lists the titles of the top 10 most cited articles. There is 3 review articles and 3 clinical studies (Cohort Study).

**Table 4:** Top 10 articles with most-number of citations on PubMed.

No.	Article title	Number of citation
1	Emerging Updates on the Posterior Cruciate Ligament: A Review of the Current Literature [4]	126
2	Tibial tubercle-posterior cruciate ligament distance: a new measurement to define the position of the tibial tubercle in patients with patellar dislocation [5]	114
3	A prospective randomized study comparing arthroscopic single-bundle and double-bundle posterior cruciate ligament reconstructions preserving remnant fibers [6]	106
4	Isolated and combined grade-III posterior cruciate ligament tears treated with double-bundle reconstruction with use of endoscopically placed femoral tunnels and grafts: operative technique and clinical outcomes [7]	101
5	Kinematic analysis of the posterior cruciate ligament, part 1: the individual and collective function of the anterolateral and posteromedial bundles (8)	95
6	Long-term results of isolated anterolateral bundle reconstructions of the posterior cruciate ligament: a 6- to 12-year follow-up study (9)	93
7	Minimum 10-year follow-up of patients after an acute, isolated posterior cruciate ligament injury treated nonoperatively (10)	92
8	Current concepts review: the posterior cruciate ligament (11)	91
9	Radiographic landmarks for tunnel positioning in posterior cruciate ligament reconstructions (12)	71
10	Anterolateral transtibial posterior cruciate ligament reconstruction combined with anatomical reconstruction of posterolateral corner insufficiency: comparison of single-bundle versus double-bundle posterior cruciate ligament reconstruction over a 2- to 6-year follow-up (13)	69

### 3.4 Country

**Fig. 2:**
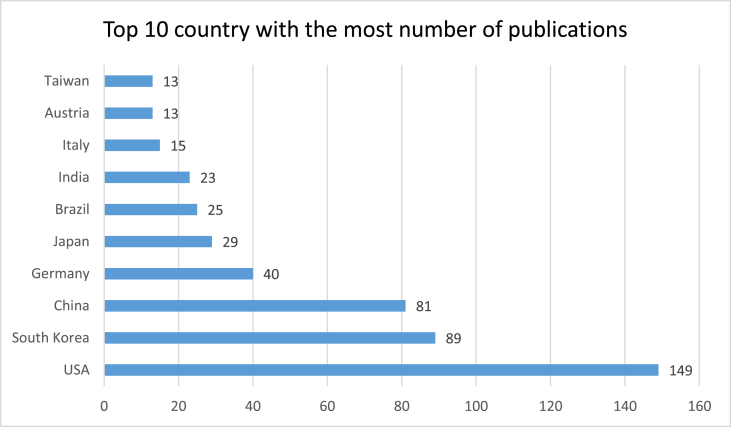
Top 10 countries most publishing on the PCL.

Fig. 2 shows the top 10 country with the most publications in ten years. The country with the most number of publication is USA. It has published 149 articles, which is constituted 25% of all articles published on this topic.

### 3.5 Timeline trends of PCL techniques and types of studies

**Fig. 3:**
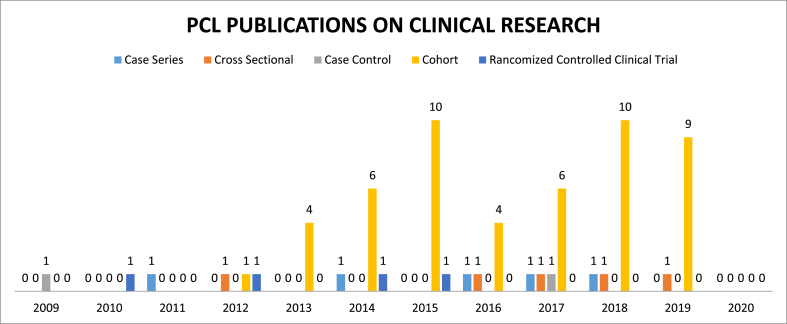
PCL publication in clinical research.

Table 5 shows the type of study by year. Most of study is clinical research (36%), article review (13%) and imaging study (12%). Studies of high quality (with higher levels of evidence) including systematic reviews, meta-analyses, and randomized studies, was showed an increment trend in the recent years.Fig. 3 shows the clinical study among 10 years.The most type of clinical study is cohort study.

**Table 5:** Trend of publication by year and type of study on PCL.

Year	Molecular/Laboratory Study	Animal Study	Cadaveric Study	Biomechanics Study	Study of Epidemiology	Imaging Study	Surgical Technique	Case Report	Clinical Research	Meta-analysis & Systematic Review	Review Article	Total
2009	0	0	5	0	0	5	2	3	16	0	4	35
2010	0	0	4	1	0	3	5	8	11	0	8	40
2011	1	2	3	1	0	1	1	5	8	0	5	27
2012	1	2	8	1	0	5	6	7	21	0	7	58
2013	3	0	12	3	0	5	3	3	25	1	8	63
2014	2	0	8	1	0	7	5	4	22	0	7	56
2015	1	1	3	5	1	7	5	4	28	1	6	62
2016	0	3	5	1	0	13	4	7	19	0	7	59
2017	0	1	4	0	1	8	8	8	21	4	10	65
2018	1	0	2	0	0	12	8	1	15	2	14	55
2019	1	2	7	1	0	7	9	10	26	1	3	67
2020	0	0	1	0	0	0	0	0	4	0	1	6
Total	10	11	62	14	2	73	56	60	216	9	80	593

## 4 Discussion

PubMed is an excellent resource for information on medical research. Until 2014, PubMed used to be updated 5 times a week. However, since June 2014, updating is done daily.

The number of publications on the PCL has increased since 2009. This number was about 35 in 2009, and the number of increment is almost twice 67 articles in 2019. Publications in recent years have had several software tools available to speed up the process of writing, editing, and accurate reporting and easy to submit in journal. These include better statistical methodologies as well as better and faster submission, reviewing, and editing processes by journals [5].

In knee injuries, posterior cruciate ligament (PCL) tears are rarer than anterior cruciate ligament tears. The previous studies have reported an incidence between 1 and 47% for PCL injuries in acute knee ligament injuries. Compare the ACL publication, about 11.940 articles in recent ten years, PCL publication only 593 articles. A few number of PCL articles were caused by the little incidence of PCL injury.

The natural history, treatment, rehabilitation protocol, and outcomes of a PCL-deficient knee have not described clearly. The appropriate treatment for isolated PCL injury remains a controversial topic in knee surgery. Commonly, nonoperative treatment of isolated PCL tears grade 1 and 2 has been recommended, and reconstruction surgery has been done for case of persistent instability, grade 3 or multiple ligament injuries [6].

All of these journal is well known in field of orthopaedic which 9 journals is Q1 and 1 journal is Q2 according to www.scimagojr.com. Table 2 shows top first author that 4 of the 10 first author were from South Korea. South Korea was also second order after America in the rank of most countries in the publication of articles (see Fig. 2). Table 3 shows the top 10 center/universities and author in all position publishing on the PCL, respectively. Among the these author affiliations, 4 of the top 10 were located in the United States of America and South Korea. One author affiliation was in Norway and the 1 last was in Austria.

Article with the title “Emerging Updates on the Posterior Cruciate Ligament: A Review of the Current Literature [7]” is the most cited article with 126 citation times. Five articles were clinical research (cohort study), 2 articles were imaging study, 2 articles were review article, and 1 article was surgical technique. United States of America had published about 25% of all articles on the PCL, followed by South Korea (15%) (Fig. 2).

Most of study is clinical research (36%) especially cohort study (Table 4 and Fig. 3) that indicates the interest in outcome follow up after surgery and rehabilitation program. Second most study was review article that discussed about current treatment or conflicting issues in PCL injury from diagnosis, treatment, and rehabilitation program. Limitations of the analysis are lot of articles data, the results may not be consistent and hence it can be inaccurate. However, trends would be similar across most databases. Whenever results were felt to be inconsistent, a search was run a minimum of several times until a consistent number was obtained. Results of mining data depend on how the data are entered in the database.

## 5 Conclusion

Mining data on the PCL in PubMed produced useful information about good sources of publications on this topic, including authors/journals that could be followed in future study. The strength of their association with other authors could potentially indicates co-workers, common research interests, and collaborative studies.

## Ethical approval

This is review article, no need ethical approval.

## Funding

The authors declare that this study had no funding resource.

## Authors' contributions

Sholahuddin Rhatomy, Dwikora Novembri Utomo, Heri Suroto, and Ferdiansyah Mahyudin conceived the study, collected the data, analysed data. Sholahuddin Rhatomy and Dwikora Novembri Utomo prepared and drafted the manuscript. Sholahuddin Rhatomy and Dwikora Novembri Utomo edited manuscript. Sholahuddin Rhatomy visualized the data into table and graph. Sholahuddin Rhatomy, Dwikora Novembri Utomo, Heri Suroto, and Ferdiansyah Mahyudin reviewed and revised the manuscript.

## Registration of research studies

This is Review article, no need registration of research studies.

## Guarantor

Sholahuddin Rhatomy,MD.

## Availability of data and material

Data will be provided by request.

## Provenance and peer review

Not commissioned, externally peer reviewed.

## Declaration of competing interest

No potential conflict of interest relevant to this article was reported.
